# Distinct Histone Modifications Modulate *DEFB1* Expression in Human Vaginal Keratinocytes in Response to *Lactobacillus* spp.

**DOI:** 10.1007/s12602-017-9286-6

**Published:** 2017-05-15

**Authors:** Jaehyouk Lee, Ara Jang, Jin Wook Kim, Jun Hyun Han, Byung Hee Chun, Hye Su Jung, Che Ok Jeon, Soon Chul Myung

**Affiliations:** 1 Department of Urology, Chung-Ang University College of Medicine, 84 Heukseok-ro, Dongjak-gu, Seoul, 06974 Republic of Korea; 2 Advanced Urogenital Diseases Research Center, Chung-Ang University College of Medicine, 84 Heukseok-ro, Dongjak-gu, Seoul, 06974 Republic of Korea; 3 Bio-Integration Research Center for Nutra-Pharmaceutical Epigenetics, Chung-Ang University, 84 Heukseok-ro, Dongjak-gu, Seoul, 06974 Republic of Korea; 4 Department of Urology, Hallym University Dongtan Sacred Heart Hospital, Hwaseong-si, 18450 Republic of Korea; 5 Department of Life Science, Chung-Ang University, 84 Heukseok-ro, Dongjak-gu, Seoul, 06974 Republic of Korea

**Keywords:** *DEFB1*, *Lactobacillus*, DNA methylation, Histone modifications, Vaginal keratinocyte

## Abstract

Vaginal commensal lactobacilli are considered to contribute significantly to the control of vaginal microbiota by competing with other microflora for adherence to the vaginal epithelium and by producing antimicrobial compounds. However, the molecular mechanisms of symbiotic prokaryotic-eukaryotic communication in the vaginal ecosystem remain poorly understood. Here, we showed that both DNA methylation and histone modifications were associated with expression of the *DEFB1* gene, which encodes the antimicrobial peptide human β-defensin-1, in vaginal keratinocyte VK2/E6E7 cells. We investigated whether exposure to *Lactobacillus gasseri* and *Lactobacillus reuteri* would trigger the epigenetic modulation of *DEFB1* expression in VK2/E6E7 cells in a bacterial species-dependent manner. While enhanced expression of *DEFB1* was observed when VK2/E6E7 cells were exposed to *L. gasseri*, treatment with *L. reuteri* resulted in reduced *DEFB1* expression. Moreover, *L. gasseri* stimulated the recruitment of active histone marks and, in contrast, *L. reuteri* led to the decrease of active histone marks at the *DEFB1* promoter. It was remarkable that distinct histone modifications within the same promoter region of *DEFB1* were mediated by *L. gasseri* and *L. reuteri*. Therefore, our study suggested that one of the underlying mechanisms of *DEFB1* expression in the vaginal ecosystem might be associated with the epigenetic crosstalk between individual *Lactobacillus* spp. and vaginal keratinocytes.

## Introduction

Emerging evidence suggests a critical role for epigenetic mechanisms, such as DNA methylation and histone modifications, in modulating the expression of host genes during bacterial infections [1]. Various bacterial products or secreted factors, including lipopolysaccharides (LPS), have been shown to provoke histone modification and chromatin remodeling of inflammatory genes, thereby resulting in transcriptional repression at a gene-specific level. There is now wide acceptance that host-bacterial interactions occur not only after pathogenic bacterial infection but also continuously between commensal bacteria and the host [2]. Indeed, commensal microbes can constitute the first line of defense against infection by participating in the maintenance of immune homeostasis through epigenetic modification of host genes. Probiotics, defined as live microorganisms that confer a health benefit on the host, have recently been found to induce antimicrobial peptides against pathogens in the gastrointestinal tract [3]. The human vagina continuously responds to immunologically unique conditions in which an efficient antimicrobial defense system is required to eliminate potential pathogens, while tolerating beneficial commensal microbes [4, 5]. The predominant bacterial species of the normal vaginal microbiota is *Lactobacillus* [5], whose major role is to inhibit pathogen colonization by direct killing or competition for host cell receptors. Lactobacilli are known to present at concentrations of 10^7^ to 10^8^ colony-forming units (CFU)/ml in the vaginal tracts of clinically healthy individuals [6]. Vaginal lactobacilli produce antimicrobial compounds such as bacteriocins and hydrogen peroxide, and they are considered the most important probiotic strains among the normal flora of healthy individuals. However, whether secretion of antimicrobial peptides such as defensins from vaginal epithelial cells is affected by exposure to *Lactobacillus* spp. in the female reproductive tract has not been determined so far.

Antimicrobial peptides (AMPs) play key roles in the innate immune responses against bacteria, viruses, fungi, and parasites [7, 8]. Defensins are small, polycationic AMPs that include α- and β-defensins and cathelicidins. Among these, human β-defensin-1 (HBD-1) is considered to be constitutively produced at low levels in various epithelial tissues, including the skin and the respiratory and urogenital tracts, with little regulation in response to infection or other stimuli [9]. The immunomodulatory function of HBD-1 is attributed to its chemotactic attraction for immature dendritic cells and memory T cells [10]. In addition, HBD-1 is recognized as a potential tumor suppressor in urological cancers [11]. Recently, we have reported that site-specific CpG dinucleotides are responsible for DNA methylation-mediated regulation of *DEFB1* expression in prostate cancer cells [12]. Furthermore, transcriptional activation of *DEFB1* is associated with specific histone modifications in bronchial epithelial cell biopsies of patients with chronic obstructive pulmonary disease [13]. In a recent report, lactobacilli and the probiotic cocktail VSL#3 strengthened the intestinal barrier functions through the upregulation of HBD-2 [14]. Moreover, epigenetic regulation of HBD-2 expression in response to oral bacteria has been found in gingival epithelial cells [15]. Additionally, several AMP genes, encoding HBD-2, HBD-3, and LL37, were induced by histone deacetylase (HDAC) inhibition in human colonic epithelial cells upon challenge with *Escherichia coli* [16]. However, the epigenetic mechanisms underlying both the constitutive and induced modulations of *DEFB1* expression in response to environmental challenges remain to be clarified. We were interested in examining if the probiotic commensal *Lactobacillus* can lead to promoter-specific modulations that fine-tune the expression of *DEFB1* in vaginal epithelial cells.

To the best of our knowledge, the present study is the first to identify the critical promoter regions of *DEFB1* that are epigenetically influenced by lactobacilli in vaginal keratinocytes. We indicate here that *Lactobacillus gasseri*, one of the most prevalent commensal species in the vagina, might enhance the expression of *DEFB1* mRNA and consequently increase the level of HBD-1 protein, whereas another commensal species *Lactobacillus reuteri* may attenuate *DEFB1* transcription through species-specific, opposing histone modifications in vaginal keratinocytes.

## Materials and Methods

### Human Cell and Bacterial Cultures

The human vaginal keratinocyte VK2/E6E7 cell line (ATCC CRL-261) was obtained from the American Type Culture Collection (Manassas, VA, USA). VK2/E6E7 cells were grown in Keratinocyte Serum Free Medium (Invitrogen, Carlsbad, CA, USA) and maintained at 37 °C in a humidified incubator at 5% CO_2_. We treated the VK2/E6E7 cells with 2′-deoxy-5-azacytidine (DAC) for 72 h and with trichostatin A (TSA) for 24 h. Each treatment with DAC or TSA was performed in at least triplicate. DAC and TSA were obtained from Sigma-Aldrich (St. Louis, MO, USA).

The bacterial strains *L. gasseri* (KCTC 3143) and *L. reuteri* (KCTC 3594) were obtained from the Korean Collection for Type Culture (Daejeon, Korea). These strains were cultured in De Man, Rogosa and Sharpe broth (Difco, Detroit, MI, USA) at 37 °C for 16 h under aerobic conditions. The optical densities of the bacterial cultures at 600 nm in the logarithmic growth phase were measured, and VK2/E6E7 cells were then exposed to 10^8^ CFU/ml of either *L. gasseri* or *L. reuteri* for 48 h. Each bacterial exposure was performed in at least triplicate.

### Quantitative RT-PCR

Total RNA was isolated from the cell lines using the RNeasy Mini Kit (Qiagen, Hilden, Germany) following the manufacturer’s instructions. cDNA synthesis for real-time two-step RT-PCR was performed with 1 μg of total RNA using the QuantiTect Reverse Transcription Kit (Qiagen), and then 1 μl of diluted cDNA was utilized for cycling reactions using the Rotor-Gene SYBR Green PCR Kit (Qiagen) according to the manufacturer’s instructions. The amplification and quantitative analysis were performed in the Rotor-Gene Q 5plex HRM system (Qiagen). Thermal cycling was performed using the default conditions of the Rotor-Gene Q Series Software (Qiagen), which consisted of 5 min at 95 °C followed by 40 rounds of 5 s at 95 °C and 10 s at 60 °C. The transcript level measured was normalized to the results of a QuantiTect Primer Assay (Qiagen) for hypoxanthine phosphoribosyltransferase 1 (*HPRT1*), which was used as an internal control.

### Immunocytochemistry

The VK2/E6E7 cells were grown on glass coverslips, fixed with 4% paraformaldehyde for 40 min, and then permeabilized with 0.1% Triton X-100 for 20 min at room temperature (RT). Cells were then blocked with 3% bovine serum albumin (Sigma-Aldrich) for 2 h to block non-specific antibody binding and then incubated overnight at 4 °C with antibody against HBD-1 (Abcam, Cambridge, UK). After washing, cells were incubated with FITC-conjugated anti-mouse IgG (1:1000 dilution; Sigma-Aldrich) for 1 h at RT, and DNA was counterstained with propidium iodide (Sigma-Aldrich). Coverslips were mounted with Fluorescence Mounting Medium (DAKO). Fluorescence was detected by confocal laser microscopy using a Zeiss LSM510 instrument (Zeiss, Jena, Germany).

### Bisulfite Sequencing

Genomic DNA was extracted from the cell lines using the QIAamp DNA Mini Kit (Qiagen). Bisulfite modification of the genomic DNA was performed using the EZ DNA Methylation-Lightning™ Kit (Zymo Research), according to the manufacturer’s instructions. The bisulfite-converted genomic DNA was amplified using either a primer set specific for the “proximal” promoter region of *DEFB1* [nucleotides −624 to −120 upstream from the transcription start site (TSS), which was denoted as +1] containing six CpG sites or a primer set specific for the “distal” promoter region of *DEFB1* (nucleotides −1000 to −600 upstream from the TSS) containing six CpG sites. The sequences of the PCR primers used were as follows: “proximal” region, forward (5′-TTGGTAGGGTTGAAGTGGGAG-3′) and reverse (5′-TAAAACCCTAATACCAACTCCTC-3′); “distal” region, forward (5′-TTAAGGAAAATTTGAGGGATATTTGG-3′) and reverse (5′-CAAATATCCCTCAAATTTTCCTTAATTC-3′). Cycling conditions were as follows: initial denaturation at 94 °C for 10 min; 45–50 cycles of denaturation at 94 °C for 30 s, annealing at 52 °C for 30 s, and extension at 72 °C for 30 s; and a final extension at 72 °C for 10 min. The PCR products were purified and subcloned into the pGEM-T Easy Vector (Promega) for subsequent sequencing. The nucleotide sequences of 20–25 independent clones were analyzed.

### Chromatin Immunoprecipitation (ChIP)

ChIP analysis was carried out using an EZ ChIP kit (Millipore, Billerica, MA, USA) following the manufacturer’s protocol. Briefly, 4 × 10^5^ VK2/E6E7 cells were plated in a 100-mm culture dish the day before bacterial exposure. After 48 h of incubation, formaldehyde-treated cells were resuspended in SDS lysis buffer, and the cell lysates were sheared by sonication. The chromatin fragments were immunoprecipitated overnight with antibodies against AcH3, H3K4me3, and H2A.Z (all antibodies from Millipore). Precipitated chromatin was then washed, reverse-crosslinked, and digested with RNase A and proteinase K. The purified DNA was analyzed by quantitative PCR using the Rotor-Gene Q 5plex HRM system (Qiagen). The sequences of the PCR primers used were as follows: “ChIP 1” region (nucleotides −320 to −186 upstream from the TSS), forward (5′-GTTTGTCTTGCAGGAAGACAATC-3′) and reverse (5′-AACAGGCAGTTCACACTGGAG-3′); “ChIP 2” region (nucleotides −500 to −392 upstream from the TSS), forward (5′-ACTTTCTGAGGAGTGCCCTTTG-3′) and reverse (5′-CCTTCTCATCTCTCCCCTTATG-3′). Thermal cycling was performed using the default conditions of the Rotor-Gene Q Series Software (Qiagen), which consisted of 5 min at 95 °C followed by 40 rounds of 5 s at 95 °C and 10 s at either 58 or 60 °C for “ChIP 1” or “ChIP 2,” respectively.

### Statistical Analysis

Data are presented as the mean ± standard error of the mean (SEM) and analyzed using Student’s *t* test. A *P* value of <0.05 was considered statistically significant.

## Results

### Epigenetic Regulation of *DEFB1* Expression in Vaginal Keratinocytes

**Fig. 1:**
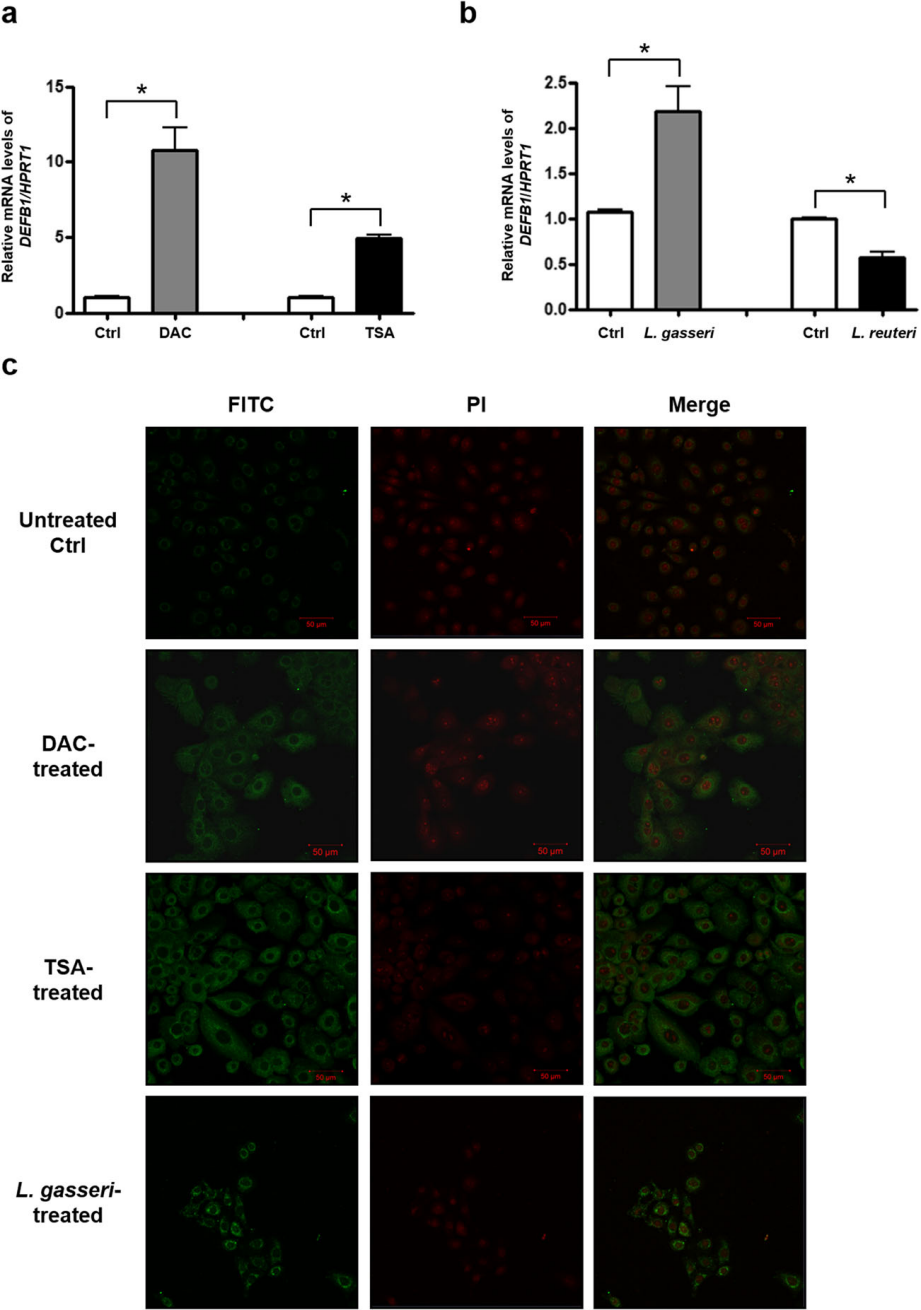
Significant changes in *DEFB1* expression in vaginal keratinocytes VK2/E6E7 after treatment with either epigenetic inhibitors or *Lactobacillus* spp. **a** Quantitative RT-PCR analysis showed the induction of *DEFB1* expression upon treatment with the DNA-demethylating agent 2′-deoxy-5-azacytidine (DAC) or the HDAC inhibitor trichostatin A (TSA). **b** Up- or down-regulation of the *DEFB1* mRNA level in response to exposure to either *L. gasseri* or *L. reuteri*. After normalization of *DEFB1* expression by use of the *HPRT1* mRNA level, the relative expression level of *DEFB1* in lactobacilli-treated cells relative to that in untreated control cells was calculated. *P < 0.05. **c** Immunocytochemical analysis was carried out to confirm the data obtained from qRT-PCR. VK2/E6E7 cells were stained with anti-HBD-1 antibody (*green*) and counterstained with propidium iodide (*red*) for DNA

To determine whether epigenetic modulation of the *DEFB1* gene occurs in human vaginal keratinocytes, we first examined the transcriptional restoration of *DEFB1* in VK2/E6E7 cells after treatment with either the DNA methyltransferase inhibitor DAC or the HDAC inhibitor TSA (Fig. 1a). Compared to the level in untreated cells, a remarkable elevation of the *DEFB1* mRNA level was observed following DAC-induced DNA demethylation and TSA-mediated histone acetylation. These data imply that transcriptional regulation of *DEFB1* in VK2/E6E7 cells is associated with epigenetic mechanisms involving both DNA methylation and histone deacetylation.

### Contrasting Changes in *DEFB1* Expression Were Observed in Vaginal Keratinocytes in Response to Two Different *Lactobacillus* spp.

To investigate changes in *DEFB1* expression in VK2/E6E7 cells following exposure to either *L*. *gasseri* or *L. reuteri*, *DEFB1* mRNA and HBD-1 protein levels were assessed. Upregulation of *DEFB1* mRNA in VK2/E6E7 cells was found in response to *L. gasseri*, but *DEFB1* mRNA was downregulated in the presence of *L. reuteri* (Fig. 1b). An immunocytochemistry analysis showed enhanced expression of HBD-1 after VK2/E6E7 cells were exposed to *L. gasseri* or treated with either DAC or TSA (Fig. 1c). These findings indicated that *L. gasseri* and *L. reuteri* modulated expression of *DEFB1*, which might undergo epigenetic regulation in VK2/E6E7 cells, in a bacterial species-dependent manner.

### Specific Regions Within the Non-CpG Island Promoter of *DEFB1* Were Affected by DNA Methylation

**Fig. 2:**
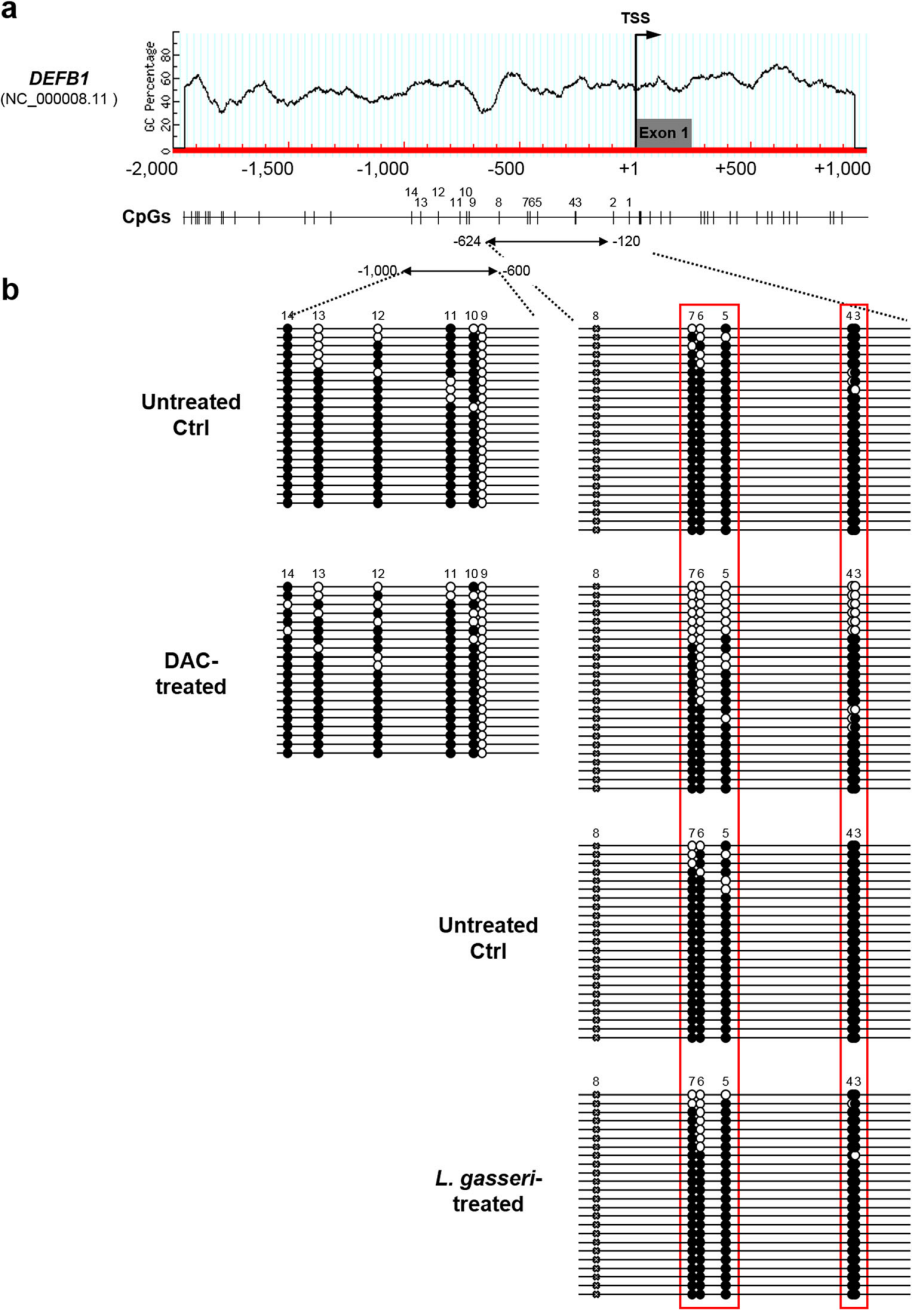
Bisulfite sequencing analysis of the *DEFB1* promoter region in VK2/E6E7 cells. **a** The schematic representation of the 5′ end of *DEFB1* is marked by the typical non-CpG island promoter. The *arrow* indicates the transcription start site (TSS), and the *gray box* represents the first exon. CpG loci and the relative positions of primers for bisulfite sequencing are indicated under the CpG island plot. **b** Bisulfite sequencing of the *DEFB1* promoter, which is divided into “proximal” and “distal” regions, was performed on VK2/E6E7 cells treated with DAC or *L. gasseri*. A single nucleotide polymorphism present in the CpG 8 locus within the *DEFB1* promoter region is denoted by the symbol *x*. *Circles* represent single CpG sites, and each row of *circles* represents the DNA sequence of an individual clone. Unmethylated and methylated CpG sites are depicted as *white* and *black circles*, respectively. The *red boxes* highlight the promoter regions spanning the key CpG loci that are important for the difference in the *DEFB1* expression in DAC-treated VK2/E6E7 cells compared to untreated control cells

To identify the site-specific CpG dinucleotides that are important for *DEFB1* expression in the DAC-treated VK2/E6E7 cells, we performed bisulfite sequencing of a region spanning 1 kb upstream from the TSS of the non-CpG island promoter of *DEFB1* (Fig. 2a), the promoter activity of which has previously been demonstrated using a reporter assay [11]. We determined the methylation profiles of the five CpG dinucleotides (CpG 3 to CpG 7) located at the “proximal” region, assigning the first CpG dinucleotide 31-bp upstream from the TSS of *DEFB1* as CpG 1, and of the six CpG dinucleotides (CpG 9 to CpG 14) located at the distal region of *DEFB1*. Since a single nucleotide polymorphism (rs2978863) is present in the CpG 8 locus within the *DEFB1* promoter region in VK2/E6E7 cells, the five CpG dinucleotides (CpGs 3 to 7) other than the CpG 8 site were subjected to bisulfite sequencing. As shown in Fig. 2b, the CpG methylation status at the “proximal” promoter region of *DEFB1* was preferentially affected by the DNA-demethylating agent DAC. However, no significant difference was observed in the methylation profile of the “distal” promoter region of *DEFB1* in the VK2/E6E7 cells treated with DAC. These results suggested that the differentially methylated CpG dinucleotides, i.e., CpG 3–CpG 7, within the *DEFB1* promoter region might be particularly important for the transcriptional regulation of *DEFB1* in VK2/E6E7 cells.

Additionally, the methylation profile of these five CpG sites within the *DEFB1* promoter region indicated a subtle difference in the VK2/E6E7 cells exposed to *L. gasseri* compared to that in the untreated control cells (Fig. 2b). Therefore, we hypothesized that altered expression of *DEFB1* in VK2/E6E7 cells in response to exposure to *Lactobacillus* spp. might be mediated via histone modifications rather than via DNA methylation.

### The Promoter Regions Corresponding to DNA Methylation-Mediated *DEFB1* Regulation Were Marked by Species-Specific, Opposing Histone Modifications in VK2/E6E7 Cells Exposed to Two Different *Lactobacillus* spp.

**Fig. 3:**
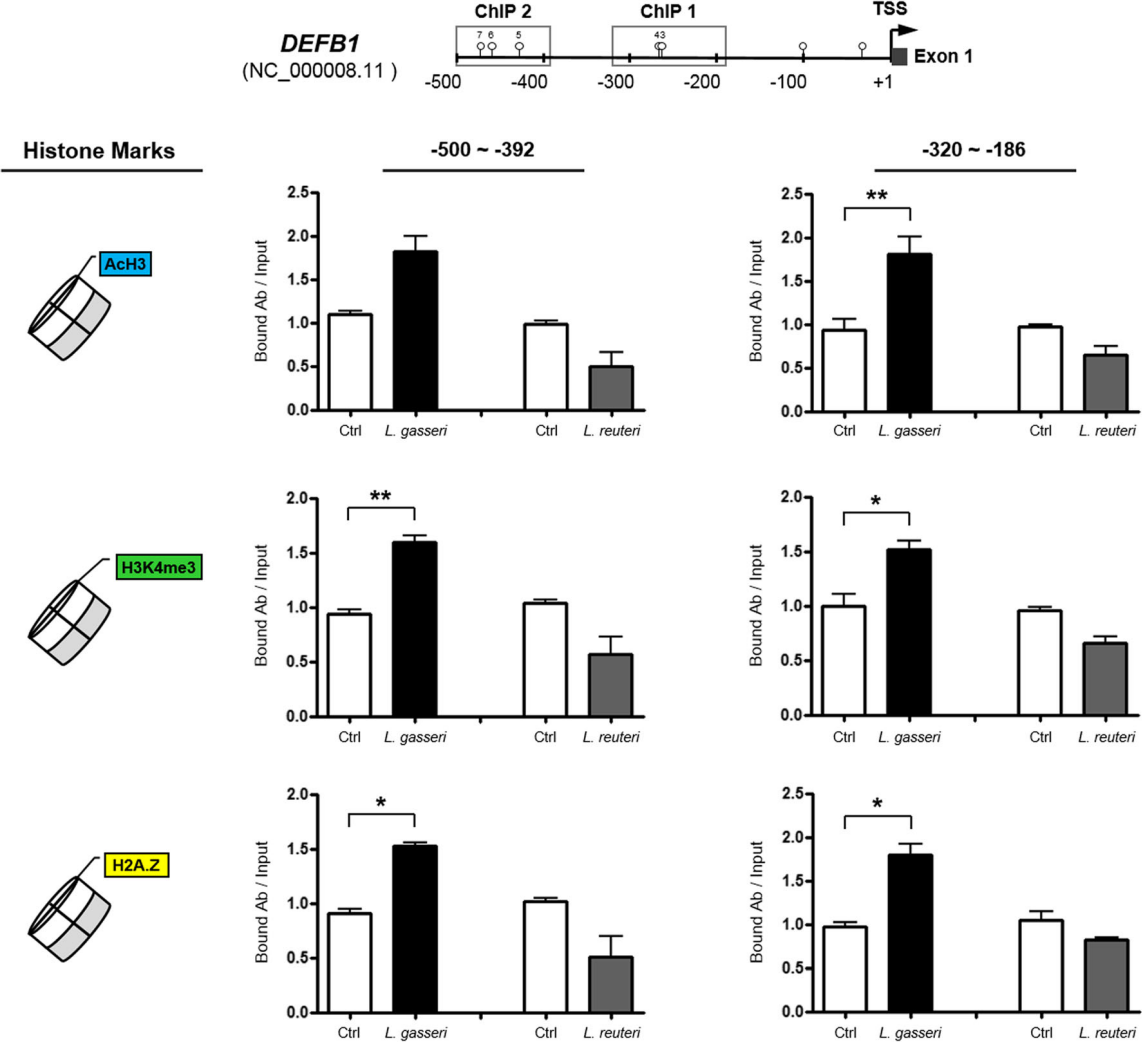
Summary of changes in histone modifications within the *DEFB1* promoter in response to either *L. gasseri* or *L. reuteri*. The amplified regions (positions −500 to −392 and −320 to −186 upstream from the *DEFB1* TSS) used for ChIP analysis with the indicated antibodies are marked by *gray boxes* (ChIP 1 and ChIP 2) and include CpGs 3 and 4 and CpGs 5, 6, and 7 (depicted as *lollipops*), respectively. While exposure to *L. gasseri* induced the enrichment of active histone marks, such as AcH3, H3K4me3, and H2A.Z, within the *DEFB1* promoter, decreases in the same histone marks were observed in the corresponding experiments for *L. reuteri*. Each experiment was repeated at least three times, and the data are presented as ratios between the immunoprecipitated DNA (bound Ab) and the input DNA (mean ± SD). *P < 0.05; **P < 0.01

To investigate the modulation of histone occupancy at the *DEFB1* promoter region in response to either *L. gasseri* or *L. reuteri*, two fragments of the proximal promoter region, spanning two CpG dinucleotides (CpGs 3 and 4; positions −320 to −186, “ChIP 1” region) and three CpG dinucleotides (CpGs 5, 6, and 7; positions −500 to −392, “ChIP 2” region), were analyzed using the ChIP assay (Fig. 3). We first tested whether the recruitment of acetylated histone H3 (AcH3) was associated with these key regions, and we observed an enrichment of AcH3 in both the “ChIP 1” and “ChIP 2” regions within the *DEFB1* promoter after exposure to *L. gasseri*, while reduction in the level of this histone mark was detected in response to *L. reuteri*. We then determined if these promoter regions were associated with changes in the level of an established mark typical for transcriptionally active chromatin, i.e., trimethylation of Lys4 of histone H3 (H3K4me3) [17, 18]. We found that levels of H3K4me3 were increased in both “ChIP 1” and “ChIP 2” regions within the *DEFB1* promoter upon exposure to *L. gasseri*, while reduction in the level of this protein was detected in response to *L. reuteri*. Finally, the deposition of the histone variant H2A.Z, which is enriched in regulatory elements at the 5′ ends of many genes and may destabilize nucleosomes to facilitate transcriptional initiation [19], was also examined. Increased H2A.Z levels in both the “ChIP 1” and “ChIP 2” regions within the *DEFB1* promoter were observed following exposure to *L. gasseri*, but a consistent decrease in the level of this histone mark was found upon exposure to *L. reuteri*. Collectively, these data indicate that these proximal promoter regions of *DEFB1* might undergo distinct *Lactobacillus* spp.-dependent histone modifications to achieve epigenetic regulation of *DEFB1* expression in VK2/E6E7 cells in a mutually exclusive manner.

## Discussion

An epigenetic trait is defined as a stably heritable phenotype resulting from changes in a chromosome without alterations in the DNA sequence [20]. Epigenetic mechanisms including DNA methylation, histone modification, and microRNA have been implicated in a huge variety of human diseases correlating with gene–environment interactions [21, 22]. Methylation of the cytosine base within a CpG dinucleotide context in gene promoters is generally associated with transcriptional repression [23, 24]. In the present study, we revealed that the molecular events underlying up- or down-regulation of *DEFB1* expression in vaginal cells were associated with both promoter CpG methylation and histone modifications. Our findings also showed that distinct species of the same genus of commensal bacteria could affect host gene (i.e., *DEFB1*) expression differently by mutually antagonistic processes involving epigenetic alterations. We noted that significant changes in histone marks occurred in the proximal promoter region of *DEFB1*, in which critical CpG sites were affected by treatment with a DNA demethylating agent, in a bacterial species-dependent manner. Moreover, our data implied that *L. gasseri* and *L. reuteri* could control *DEFB1* expression by modulating the recruitment of active histone marks within the *DEFB1* promoter region.

Genus *Lactobacillus* represents a large number of species and strains of lactic acid bacteria (LAB) that produce lactic acid as the main end-product of carbohydrate metabolism [25]. There has been impressive achievement of studies demonstrating that probiotic lactobacilli play a vital role in host physiology, including the digestion and assimilation of nutrients, protection against pathogen colonization, and modulation of immune responses [26]. Potential antimicrobial activity of these microbes can be characterized by production of different antimicrobial metabolites [27]. It has been well established that antimicrobial substances produced by LAB can be divided into two main groups: low molecular mass substances (<1000 Da) and high molecular mass substances (>1000 Da), such as bacteriocins. LAB bacteriocins were shown to have potential not only as antibacterial but also as antiviral and anticancer agents [28]. Recently, gassericin E (GasE), a novel bacteriocin produced by *L. gasseri* EV1461, was characterized as a vaginal probiotic candidate [29]. In the present study, we might pave the way for the possibilities of antimicrobial action of probiotic lactobacilli, which could modulate expression of host-derived factors in the vaginal keratinocytes.

The tissues of the female reproductive tract are vulnerable to a large number of infectious agents, and the stratified squamous epithelium of the vagina represents a physical barrier to pathogens. Mucosal surfaces of the vaginal tract are equipped with a homeostatic balance between immunity to harmful pathogens and tolerance for maintaining useful bacteria that represents a unique regulatory challenge for the mucosal immune system [4]. In addition, the indigenous microbiota of the vaginal tract constitute an important defense mechanism to avoid invasion and colonization by foreign pathogenic microbes. Recent findings have revealed an important function of the commensal microbiota in protecting the host from infection [30]. The composition of individual species of the microbiota has the potential to modulate immune homeostasis [31], which in turn may affect the susceptibility of the female reproductive tract to infection. An emerging body of evidence has indicated that epigenetic events play a key role in host–microorganism interactions in infectious diseases [1]. Recent evidence has consolidated the notion that intestinal commensal microbiota play a critical role in the modulation of mucosal immune homeostasis [31, 32]. Further, one study has shown that DNA methylation of the *TLR4* gene might be affected by commensal bacteria in the large intestine of mice [33]. In addition, one previous report highlighted that HDAC3 was responsible for the coordination of commensal-bacteria-dependent intestinal homeostasis [34], suggesting that altered histone acetylation of host genes might occur in the presence of commensal bacteria. To date, there has not been any evidence suggesting that the unique epigenetic context at a CpG-poor promoter might also contribute to the commensal species-specific control of host gene expression related to innate immune responses. Our findings raise the novel possibility that distinct species of the same commensal *Lactobacillus* genus may have developed molecular mechanisms by which the expression of the host gene *DEFB1* can be modulated by species-specific histone marks in mutually exclusive ways. Additionally, the overexpression of AMPs in vaginal fluid has been detected during infections of the female genital tract [35]. For instance, HBD-2 can be induced by *Candida albicans* [36], and both HBD-2 and -3 show an overexpression during *Chlamydia trachomatis* or *Neisseria gonorrhoeae* infections [37]. Our ongoing investigation on epigenetic modulation of these defensins after exposure to commensal lactobacilli, including *L. gasseri* and *L. reuteri*, could help better understand the inducible changes in AMPs in the vaginal ecosystem. Furthermore, genomic and empirical evidence supports the probiotic application of *L. gasseri* for maintenance of vaginal homeostasis [38]. We anticipate that more personalized probiotics or prebiotic mixtures derived from symbiotic *Lactobacillus* spp. may help establish and maintain a healthy vaginal ecosystem that is beneficial for host innate immunity.

In conclusion, our findings provided the novel evidence that the specific promoter regions of *DEFB1* might be affected by commensal lactobacilli through species-specific, opposing histone modifications in vaginal keratinocytes.
